# OddCAPS: a simple, low-cost, universal technique for detecting single nucleotide variants

**DOI:** 10.64898/2026.07.16.738826

**Published:** 2026-08-11

**Authors:** Karin Kawaguchi, Yuzuha Komachiya, Mai Muto, Ryoka Teshima, Naoko Sakai, Hayao Ohno

## Abstract

Derived Cleaved Amplified Polymorphic Sequences (dCAPS) assays have been widely performed historically to detect known base substitutions in many model organisms—notably *Caenorhabditis elegans* , *Arabidopsis thaliana* , and yeast—where chemical mutagens that induce point mutations are frequently used. With the rise of whole-genome sequencing and genome editing technologies, dCAPS is increasingly applied to detect diverse nucleotide changes across a broader range of species. However, a key limitation of dCAPS is that genomic target sites amenable to primer designs that both preserve PCR amplification and create recognition sites for inexpensive, high-performance restriction enzymes are scarce. Here we report One-step dual-primer dCAPS (OddCAPS), a modification that uses three primers in one reaction to overcome this constraint. Two of these primers, an intermediate primer and a dCAPS primer, sequentially introduce 1–2 base substitutions each into the amplicon, enabling up to four engineered base changes near the nucleotide of interest. By using the intermediate primer at 1/10–1/100 the concentration of the other primers, the desired product is generated directly in a single-tube, one-step PCR. Increasing the number of engineered substitutions improves the chance of using a researcher’s preferred restriction enzyme. In principle, having eight common restriction enzymes (BamHI, EcoRI, NheI, SalI, BglII, ClaI, HindIII, and MluI) suffices to detect any single-nucleotide variant in any biological or synthetic DNA sequence with this approach.

## Full Text Availability

The license terms selected by the author(s) for this preprint version do not permit archiving in PMC. The full text is available from the preprint server.

