# Supplementary material for: OddCAPS: a simple, low-cost, universal technique for detecting single nucleotide variants
Source: bioRxiv. 2026 Jul 16:2026.07.16.738826. Preprint. [Version 1] doi: 10.64898/2026.07.16.738826 (PMC13405306; doi:10.64898/2026.07.16.738826)
Supplement: 1 [file NIHPP2026.07.16.738826v1-supplement-1.pdf]

bioRxiv preprint doi: https://doi.org/10.64898/2026.07.16.738826; this version posted July 16, 2026. The copyright holder for this preprint (which was not certified by peer review) is the author/funder, who has granted bioRxiv a license to display the preprint in perpetuity. It is made available under a CC-BY 4.0 International license.

Table S1:

Strains used in this study.

C. elegans strains		
Strain	Genotype	Source
N2	*C. elegans* wild type	CGC
CAT117	*Ex*[*myo-3p::venus*].	(Chino et al., 2026)
VC20536	Whole-genome sequenced strain (*dock-11(gk126522) I; pptr-1(gk257890) V: sulp-7(gk293386) X.*)	CGC
CAT98	*pptr-1(dog3) F36G9.13(dog4)* V.	(Muto et al., Unpublished)
KQ1564	*sgk-1(ft15)* X.	CGC
JN1239	*plc-1(pe1237)* X.	(Kunitomo et al., 2013)
VC40176	Whole-genome sequenced strain (*pptr-1(gk499134)* V.)	CGC
CB4088	*him-5(e1490)* V.	CGC

bioRxiv preprint doi: https://doi.org/10.64898/2026.07.16.738826; this version posted July 16, 2026. The copyright holder for this preprint (which was not certified by peer review) is the author/funder, who has granted bioRxiv a license to display the preprint in perpetuity. It is made available under a CC-BY 4.0 International license.

Table S2: Primers used in this study.

Primer name	Sequence (5'–3', mismatches are underlined)	Target allele	Target base	Enzyme
dog3_dCAPS	TTCTTCATCGTATTTACGGAAAATTTTTGGGAATT	*pptr-1(dog3)*	C (WT)	EcoRI (GAATTC)
dog3_intermediate	TTCTTCATCGTATTTACGGAAAATTTTTGGGCATT			
dog3_R	GCCATTTTTTAGCGATTTTTCAGCGA			
dog4_dCAPS	TCGGTTCCGGTTCTCGAGCAGTCTGCATGTCCTAG	*F36G9.13 (dog4)*	G (WT)	EcoT14I (CCWWGG)
dog4_intermediate	TCGGTTCCGGTTCTCGAGCAGTCTGCATGTTCTAG			
dog4_R	TTCCATCCTGGGAATTATCGC			
gk126522_dCAPS	ACACTATCTGGATGGTCAAAAAGTGGAGCAGGAATT	*dock-11(gk126522)*	C (WT)	EcoRI (GAATTC)
gk126522_intermediate	ACACTATCTGGATGGTCAAAAAGTGGAGCAGAAACT			
gk126522_R	ATCATTTTGCTCTGCCTCTCCA			
gk257890_dCAPS	GAGCCATTTTCGGGTTTTTCTTGCATTTCCCATAT	*pptr-1(gk257890)*	G (WT)	NdeI (CATATG)
gk257890_intermediate	GAGCCATTTTCGGGTTTTTCTTGCATTTCCAATCT			
gk257890_R	ATTCCGTGTACTTCCACCATC			
gk293386_dCAPS	GAAAAAGAAGCAAAACCAATCATGGAAAAATTGAATT	*sulp-7(gk293386)*	C (WT)	EcoRI (GAATTC)
gk293386_intermediate	GAAAAAGAAGCAAAACCAATCATGGAAAAATTGTATT			
gk293386_R	GAATACCGACAGTGACACCACC			
gk499134_dCAPS	TTTTTCGGTTTTTCATCTAGAAAATCCAAAAGGATC	*pptr-1(gk499134)*	C (*gk499134*)	BamHI (GGATCC)
gk499134_intermediate	TTTTTCGGTTTTTCATCTAGAAAATCCAAAAGAATC			
gk499134_R	CGATATTTTCAGGCGCATCAAC			
pe1237_dCAPS	GACGGCCGAGCAGCCCACAAGCAATCCCCGGAATT	*plc-1(pe1237)*	C (WT)	EcoRI (GAATTC)
pe1237_intermediate	GACGGCCGAGCAGCCCACAAGCAATCCCCGGACTT			
pe1237_R	TCCACCAATTTGACCACTGA			
ft15_dCAPS	GCAAATCCTTATGGCCAAAACATACGTTTTCGACT	*sgk-1(ft15)*	C (WT)	HinfI (GANTC)
ft15_intermediate	GCAAATCCTTATGGCCAAAACATACGTTTTCGTCT			
ft15_R	CAATGATTACAGTGAGGATGTC			
e1490_dCAPS	TGAGGAATACTTGGAGTTAGCTTTCCGGAGCAGATC	*him-5(e1490)*	T (*e1490*)	BglII (AGATCT)
e1490_intermediate	TGAGGAATACTTGGAGTTAGCTTTCCGGAGCTGAGC			
e1490_R	ACAAGACGAAGTAGTGAATGAAGC			

bioRxiv preprint doi: https://doi.org/10.64898/2026.07.16.738826; this version posted July 16, 2026. The copyright holder for this preprint (which was not certified by peer review) is the author/funder, who has granted bioRxiv a license to display the preprint in perpetuity. It is made available under a CC-BY 4.0 International license.

Supplemental text:

Protocol from primer design through genotype detection by gel electrophoresis

A. Primer design and preparation

As an example, we sought to distinguish the wild-type *C. elegans* allele from the *pptr-1(gk257890)* mutant (Fig. 3A). The sequence on chromosome V that corresponds to positions 16,359,127–16,359,141 (presented here in the orientation opposite to the gene) is 5'-CCAACCT**G**TAAATGT-3' in the wild type. In the *pptr-1(gk257890)* allele, the central guanine (G) is substituted by adenine (A), producing 5'-CCAACCT**A**TAAATGT-3' (Fig. 3A).

1. Open dCAPS Finder 2.0 (Neff et al., 2002; http://helix.wustl.edu/dcaps/). Enter the wild-type sequence "CCAACCTGTAAATGT" into the field labeled "Enter the Wild Type Sequence", and enter the mutant sequence "CCAACCTATAAATGT" into "Enter the Mutant Sequence". Set "How many mismatches in the primer?" to "0" and click "submit".

Note: The ApE program (Davis & Jorgensen, 2022; https://jorgensen.biology.utah.edu/wayned/ape/) also includes a comparable "dCAPS calculator" function.

2. If you are fortunate to find a suitable restriction enzyme that you routinely use, perform PCR-RFLP (CAPS) with that enzyme. If no satisfactory enzyme is returned, go back to the first dCAPS

bioRxiv preprint doi: https://doi.org/10.64898/2026.07.16.738826; this version posted July 16, 2026. The copyright holder for this preprint (which was not certified by peer review) is the author/funder, who has granted bioRxiv a license to display the preprint in perpetuity. It is made available under a CC-BY 4.0 International license.

Finder page, increase the value of "How many mismatches in the primer?" to "1", and press "submit".

3. If you are fortunate enough to identify a satisfactory restriction enzyme and the corresponding "PRIMER SEQUENCE" does not contain a mismatch at the 3′-terminal nucleotide (underlined in the output), perform the dCAPS assay using that enzyme and primer. If no suitable enzyme is found, return to the initial screen, set "How many mismatches in the primer?" to "2", and click "submit".

Note: The 3′-terminal nucleotide is important for efficient PCR amplification; it is advisable to avoid introducing mismatches at that position.

4. Continue increasing the number of primer mismatches one increment at a time until a satisfactory restriction enzyme is identified. If the number of mismatches reaches two or greater, proceed using the OddCAPS approach described below. If permitting three mismatches still fails to identify an effective primer/enzyme pair, select one of the restriction enzymes listed in Figure 4 and design a dCAPS primer that incorporates the four specific mismatches indicated for that enzyme (see Figure 4).

Note: As mentioned in the main text, a prototype version of "OddCAPS finder," a web browser-based program designed to assist in primer design for OddCAPS, is available at the following page (Sakai et al., manuscript in preparation):

https://mcm-www.jwu.ac.jp/~onoh/OddCAPS.html

bioRxiv preprint doi: https://doi.org/10.64898/2026.07.16.738826; this version posted July 16, 2026. The copyright holder for this preprint (which was not certified by peer review) is the author/funder, who has granted bioRxiv a license to display the preprint in perpetuity. It is made available under a CC-BY 4.0 International license.

5. When two or more mismatches are necessary, implement the OddCAPS approach. Design a dCAPS primer of approximately 35 nucleotides in length that introduces 2–4 mismatches relative to the genomic template within the region spanning the fifth to the second nucleotide from the 3′ end, so that PCR amplification will create a restriction-enzyme recognition site (Figure 2A). The primer's terminal five nucleotides (for a 6-base cutter), together with the targeted base, should constitute the restriction-enzyme recognition sequence; when the target base is substituted, the site will be absent and the PCR product will therefore be resistant to digestion.

6. Design an Intermediate primer that serves as a stepping-stone between the genomic sequence and the dCAPS primer. Design the Intermediate primer so that it differs from the genomic DNA by 1–2 nucleotides, and differs from the dCAPS primer by an additional 1–2 nucleotides, thereby increasing mismatches incrementally. For the intermediate primer, avoid introducing mismatches in the two terminal nucleotides at the 3′ end, since these bases may be important for efficient PCR initiation. Place the mismatch at the second nucleotide from the 3′ end in the dCAPS primer rather than in the intermediate primer (Figs. 2A and 3A).

Note: Although untested, an intermediate primer with a slightly truncated 5′ end is unlikely to prevent PCR amplification and subsequent genotyping. In contrast, a longer dCAPS primer can improve the visual distinction between cut and uncut fragments following restriction digestion; this can allow the use of lower-concentration agarose gels, potentially resulting in further cost savings.

7. Design a Reverse primer in the antisense orientation located approximately 80–150 bp downstream of the dCAPS primer. Unlike the dCAPS or Intermediate primers, the reverse primer's

bioRxiv preprint doi: https://doi.org/10.64898/2026.07.16.738826; this version posted July 16, 2026. The copyright holder for this preprint (which was not certified by peer review) is the author/funder, who has granted bioRxiv a license to display the preprint in perpetuity. It is made available under a CC-BY 4.0 International license.

exact placement is flexible; aim for a primer 20–25 nt in length with a GC content near 40–60%. To maximize the likelihood of successful PCR, employ primer-design tools (for example, Primer-BLAST at https://www.ncbi.nlm.nih.gov/tools/primer-blast/index.cgi).

8. Confirm that the restriction site for your selected enzyme is not located in the vicinity of the target base. A proximal recognition sequence can cause the PCR amplicon to be cut irrespective of the allelic state, preventing reliable genotyping. If such a nearby site is present, choose a different restriction enzyme.

9. Synthesize the dCAPS, Intermediate, and Reverse primers through a commercial oligonucleotide synthesis service (e.g., Integrated DNA Technologies). Resuspend each lyophilized oligo to a final concentration of 100 μM in water or TE buffer.

B. Worm lysis

Note: The following worm-lysis procedure is a modification of the method of Wicks et al. (Wicks et al., 2001) for genotyping *Caenorhabditis elegans*. For other organisms, standard genotyping methods (for example, mouse tail biopsy) may be substituted.

1. Prepare a single-worm lysis buffer composed of 50 mM KCl, 10 mM Tris-HCl (pH 8.3), 2.5 mM $MgCl_2$, 0.45% NP-40, 0.45% Tween 20, and 0.01% gelatine. Sterilize the buffer by autoclaving.

bioRxiv preprint doi: https://doi.org/10.64898/2026.07.16.738826; this version posted July 16, 2026. The copyright holder for this preprint (which was not certified by peer review) is the author/funder, who has granted bioRxiv a license to display the preprint in perpetuity. It is made available under a CC-BY 4.0 International license.

2. Just before use, add 3 µL of 20 mg/mL proteinase K to 1 mL of single-worm lysis buffer to achieve a final concentration of 60 µg/mL.

3. Dispense 10 µL of single-worm lysis buffer (+ proteinase K) into each well of a 96-well PCR plate or into individual PCR tubes.

4. Using a stereomicroscope, transfer one worm to be genotyped (if you plan to use the progeny after genotyping, transfer a parent worm isolated on a plate and allowed to lay eggs overnight) into each well, taking care not to touch the sidewall of the well when depositing the worm.

5. Seal the plate carefully with PCR-grade adhesive film to prevent evaporation.

6. Lyse the worms in a thermocycler using the following program: 60°C for 60 min; 95°C for 15 min; hold at 15°C. The crude DNA lysate can be stored at 4°C for several days or at −20°C for several months.

C. PCR

1. While gently mixing by pipetting, transfer 1 µL of each crude DNA lysate from section B into individual wells of a separate 96-well PCR plate.

2. Add 10 µL of the following PCR mix to each well.

PCR mix (per sample, total: 10 µL)

bioRxiv preprint doi: https://doi.org/10.64898/2026.07.16.738826; this version posted July 16, 2026. The copyright holder for this preprint (which was not certified by peer review) is the author/funder, who has granted bioRxiv a license to display the preprint in perpetuity. It is made available under a CC-BY 4.0 International license.

- H_2O: 4.85 μL
- 2× Quick Taq HS DyeMix (TOYOBO, Cat. No. DTM-101): 5 μL
- 100 μM dCAPS primer: 0.05 μL
- 10 μM (or 1 μM) Intermediate primer: 0.05 μL
- 100 μM Reverse primer: 0.05 μL

Note: An alternative product comparable to Quick Taq is GoTaq® Green Master Mix (Promega, Cat. No. M7123).

3. Seal the plate carefully with PCR-grade adhesive film to prevent evaporation.

4. Run the following thermal-cycler program: 94°C for 2 min; 40 cycles of 94°C for 30 sec, 58°C for 30 sec, 68°C for 20 sec; 68°C for 2 min; 15°C hold.

Note: We have not experimentally verified the benefit, but we employ a slightly elevated cycle number to offset a potential slow PCR ramp-up.

Note: The post-PCR reaction solution is stable at 4°C for several days and can be stored at −20°C for extended periods.

D. Restriction enzyme digestion

1. Add 10 μL of the following restriction cocktail to each PCR well.

Restriction cocktail (per sample, total 10 μL)

bioRxiv preprint doi: https://doi.org/10.64898/2026.07.16.738826; this version posted July 16, 2026. The copyright holder for this preprint (which was not certified by peer review) is the author/funder, who has granted bioRxiv a license to display the preprint in perpetuity. It is made available under a CC-BY 4.0 International license.

- H_2O: 7.7 µL
- 10× restriction enzyme buffer (appropriate for the enzyme): 2.0 µL
- Restriction enzyme: 0.3 µL

2. Seal the plate with a fresh PCR adhesive film to prevent evaporation, briefly tap to mix, and incubate at the enzyme's recommended reaction temperature for several hours to overnight.

E. Electrophoresis

1. Apply the restriction enzyme reaction mixtures directly to a 3.5% MetaPhor™ agarose gel cast in TAE buffer and resolve the fragments by standard gel electrophoresis. When running large numbers of samples, consider using a high-throughput gel platform that accepts direct loading from 8- or 12-channel pipettes (e.g., Nihon Eido NB-1017B).

2. Stain the gel in TAE buffer containing 0.5 µg/mL ethidium bromide and visualize bands using a UV transilluminator.

Frequently Asked Questions

1. Can costs be reduced further?

Possible cost-reduction strategies include: substituting TBE for TAE to allow use of lower-percentage agarose gels; casting thinner gels to decrease agarose consumption; adopting multi-

bioRxiv preprint doi: https://doi.org/10.64898/2026.07.16.738826; this version posted July 16, 2026. The copyright holder for this preprint (which was not certified by peer review) is the author/funder, who has granted bioRxiv a license to display the preprint in perpetuity. It is made available under a CC-BY 4.0 International license.

sample high-throughput electrophoresis platforms; shortening the Intermediate primer to reduce oligonucleotide synthesis expense; lengthening dCAPS primers at the 5' end to permit use of lower-concentration agarose gels; and preparing Taq polymerase in-house rather than purchasing commercial enzyme. As an optional, modest additional step, one can also heat the used agarose gel so that it melts again, pass it through a paper filter (for example, kitchen oil-filter paper), and then allow it to solidify again for reuse (http://rizo-inc.cocolog-nifty.com/blog/2011/09/post-85ef.html).

2. Which allele should be designed to be cleavable, wild-type or mutant?

It depends on your objective. In general, it is better to design the assay so that the allele whose false-positive identification would be most problematic is the one that is cleaved. This is because failure to observe cleavage can result from technical error (for example, forgetting to add the restriction enzyme). For example, if your aim is to isolate mutant homozygotes after a cross, it is better to introduce a restriction site only in the mutant DNA to minimize false positives. Conversely, if you wish to select wild-type homozygotes (i.e., remove the mutation), it is better to produce a site that is cleaved in the wild-type DNA.

3. How can one resolve DNA fragments of roughly 100 bp by electrophoresis?

We have obtained reliable separation using high-percentage agarose gels, for example 3.5% MetaPhor™ agarose (Lonza, Cat. No. 50180) or 5% NuSieve™ 3:1 agarose (Lonza, Cat. No. 50090). Polyacrylamide gel electrophoresis is also a suitable alternative.

bioRxiv preprint doi: https://doi.org/10.64898/2026.07.16.738826; this version posted July 16, 2026. The copyright holder for this preprint (which was not certified by peer review) is the author/funder, who has granted bioRxiv a license to display the preprint in perpetuity. It is made available under a CC-BY 4.0 International license.

4. Which DNA polymerase should be used for PCR?

We recommend using a conventional, inexpensive Taq DNA polymerase rather than a high-performance, proofreading enzyme. Standard Taq is cost-effective, its reaction buffer is less likely to contain components that inhibit downstream restriction digests, and its lack of 3'→5' exonuclease (proofreading) activity reduces the chance that primer-introduced mismatches will be corrected during amplification.

5. Which Intermediate primer concentration is preferable, 1/10 or 1/100?

We use a 1:10 dilution of the Intermediate primer when the design introduces a total of 3–4 mismatches; the higher primer concentration helps ensure robust amplification under these more challenging conditions. When only 1–2 mismatches are introduced, reduce the Intermediate primer to a 1:100 dilution to promote complete restriction-enzyme digestion of the PCR product.

6. What should I do if PCR fails to yield the expected product?

Although we have not observed amplification failures with OddCAPS in our laboratory, some genomic loci may be refractory to PCR. Rather than extensive empirical optimization (e.g., iteratively varying annealing temperature or primer concentration), we recommend two-step PCR (see Discussion).
